# Resource Efficient Hardware Architecture for Fast Computation of Running Max/Min Filters

**DOI:** 10.1155/2013/108103

**Published:** 2013-10-30

**Authors:** Cesar Torres-Huitzil

**Affiliations:** Information Technology Laboratory, CINVESTAV, Km. 5.5 Carretera Ciudad Victoria-Soto La Marina, 87130 Ciudad Victoria, TAMPS, Mexico

## Abstract

Running max/min filters on rectangular kernels are widely used in many digital signal and image processing applications. Filtering with a *k* × *k* kernel requires of *k* ^2^ − 1 comparisons per sample for a direct implementation; thus, performance scales expensively with the kernel size *k*. Faster computations can be achieved by kernel decomposition and using constant time one-dimensional algorithms on custom hardware. This paper presents a hardware architecture for real-time computation of running max/min filters based on the van Herk/Gil-Werman (HGW) algorithm. The proposed architecture design uses less computation and memory resources than previously reported architectures when targeted to Field Programmable Gate Array (FPGA) devices. Implementation results show that the architecture is able to compute max/min filters, on 1024 × 1024 images with up to 255 × 255 kernels, in around 8.4 milliseconds, 120 frames per second, at a clock frequency of 250 MHz. The implementation is highly scalable for the kernel size with good performance/area tradeoff suitable for embedded applications. The applicability of the architecture is shown for local adaptive image thresholding.

## 1. Introduction

Running max/min filtering is an important operation that aims at selecting the maximum or minimum value from a set of signal elements. A window moves over all data items and at each point the max/min value of the data within the window is taken as output [1]. Max/min filters are widely used in tasks such as noise filtering, adaptive control, pattern recognition, and speech and image processing. The max filter, in gray-level image morphology [2], corresponds to the dilation operator over images using a flat structuring element (SE) or kernel, and the min filter corresponds to the erosion operator. These filters are very attractive since their computation requires only comparisons and no other arithmetic operations and because of their robust behavior in the presence of noise and signal nonstationarities [3–5].

For some image-based industrial applications, such as granulometries, particle size distribution, or local adaptive binarization, the filtering of high resolution images with large two-dimensional kernels could be very time consuming. A direct evaluation of such filters leads to *O*(*k* ^2^) comparisons per sample, being *k* the size of the kernel. A possible alternative to speed up computations is to decompose large kernels into linear or simpler ones [6]. Then, linear filtering might be implemented by efficient algorithms [7] and/or by dedicated hardware structures. Under this approach, the HGW algorithm is a widely used method to compute max/min with linear kernels whose complexity is independent of the filter size [8, 9]. Motivated by the advantages of kernel decomposition, the existence of efficient one-dimensional filtering algorithms, and by the challenge to handle the computational cost and memory requirements, the design of an architecture for fast computation of running max/min image filters with arbitrary-length rectangular kernels is presented herein. This paper proposes an efficient coarse-grain pipelined implementation of the HGW algorithm as a building block with a memory usage improvement based on distributed memory available on FPGAs compared to previous architectures that use dedicated embedded Block Ram memory.

Running max/min filters have been realized in different implementation media such as very large scale integration (VLSI) circuits and FPGAs. Most of these hardware implementations target rather small rectangular kernels and employ a pipeline technique in which a raster-scan image is sequentially fed into a long delay line and then into an array of neighboring processing elements (PEs) and the max/min operations are carried out in parallel [10, 11]. The strength of such architectures is that they can be pipelined down to a single compare-swap stage yielding high throughput and frequency. Techniques to decrease the number of comparators required to support large SEs were further introduced. For instance, in [12], authors propose a partial-result-reuse (PRR) architecture for gray-level morphological operations with flat SEs. Partial results generated during computations are kept and reused in this architecture to reduce hardware cost. However, a considerable cost, for computation and storage, is still needed for large kernels.

In [13], authors present an efficient hardware architecture to achieve erosion/dilation with very large linear kernels based on a slightly modified HGW algorithm. They propose a block mirroring scheme to suppress the need for backward scanning, to ease data propagation and memory access and to minimize memory consumption. However, embedded memories represent a large part of their design. When synthesized on a Virtex 4LX60 device, the architecture uses 3 Block Rams of 18 Kbits and 700 slices. The maximum kernel size that could be supported is 1023 over a line length of 65535 pixels. The design memory consumption is image size independent, but increasing further the parallelism, for instance, to process several image lines concurrently, is limited due to the number of Block Rams available on FPGAs. In [14], another implementation of erosion/dilation based on SE decomposition and/or efficient 1-D algorithms is proposed. The method is based on a recursive morphological decomposition of 8-convex SEs by using only causal two-pixel SEs. The proposed architecture is generic and fully regular, built from elementary interconnected modules. It has been synthesized into an FPGA, achieving high operation frequencies for any shape and size of SE; however, for large SEs a long pipeline is required.

Although some architectures for max/min filters have been developed, improvements are still needed for filtering high definition video streams in real time. On one hand, embedded memories represent a considerable cost of previous designs limiting FPGA deployment in embedded environments. One the other hand, the scalability is another concern since the architectures need major modifications when the kernel size increases. This is the primary motivation for the proposed optimized implementation, which relies on some architectural techniques used in [13].

The rest of the paper is organized as follows. The HGW algorithm is presented in Section 2. In Section 3, the proposed architecture is presented in detail as well as the strategy for mapping memory requirements to on-chip resources.  Section 4 presents the FPGA implementation, experimental results, and local adaptive thresholding as a case of study. Concluding remarks and future work are presented in Section 5.

## 2. van Herk/Gil-Werman Algorithm

The one-dimensional version of a running max filter of order *k* can be formulated as follows. Giving an input sequence of size *M*, *f*_0_, *f*_1_, *f*_2_,…, *f*_*M*−1_, the response *r*_*i*_ of the filter for *i* = 0,…, *M* − 1 is given by the following equation:

(1) $$\begin{array}{c} \;r_{i}=\underset{0\leq j<k}{\max}f_{i+j}. \end{array}$$

In the actual processing of the sequence, the boundaries usually receive some special treatment, for example, padding, periodic condition, and so forth.

**Figure 1:**
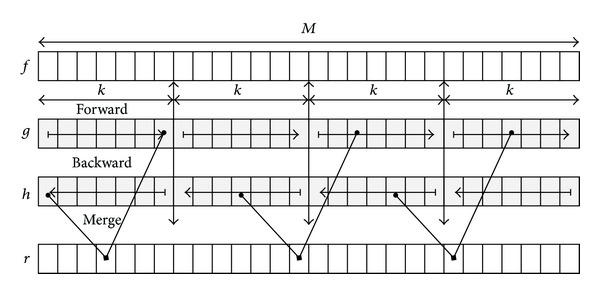
A simplified view of the main steps, forward, backward, and merge, of the HGW algorithm for a linear kernel of size *k* over an input sequence *f* of size *M*.

The HGW algorithm consists of three main processing steps [8, 9] as illustrated in Figure 1. First, the input sequence is split into segments of length *k*, where a propagation in a forward way of the current max value of *f*, for *x* = 0,…, *M* − 1, is done using the following equation:

(2) $$\begin{array}{c} g\left(x\right)=\{\begin{array}{cc} f\left(x\right), & \text{if}\;x\mathrm{mod}k=0,\\ \max\left(g\left(x-1\right),f\left(x\right)\right), & \text{otherwise}. \end{array} \end{array}$$

In a second processing step, a propagation in a backward way of the current max value of *f* is performed using the following equation:

(3) $$\begin{array}{c} h\left(x\right)=\{\begin{array}{cc} f\left(x\right), & \text{if}\;x\mathrm{mod}\left(k-1\right)=0,\\ \max\left(h\left(x-1\right),f\left(x\right)\right), & \text{otherwise}, \end{array} \end{array}$$

for *x* = *M* − 1,…, 0. Note that the values in a given segment are scanned in a reverse order to produce *h*(*x*) as opposed to *g*(*x*).

 In the last processing step, the max (or min) is computed by merging the *g* and *h* arrays using the following equation:

(4) $$\begin{array}{c} r\left(x\right)=\max\left(g\left(x+\frac{k}{2}\right),h\left(x-\frac{k}{2}\right)\right),\;x=0,\ldots ,M-1. \end{array}$$

Equations (2), (3), and (4) each require only a single comparison per array element, that is, three comparisons per sample to filter with a linear kernel of any size. The *d*-dimensional max/min filter can be computed using a kernel decomposition approach by sequentially applying the one-dimensional filter *d* times. In the two-dimensional case, only 6 comparisons per pixel are required by applying the one-dimensional HWG consecutively to rows and columns of the input image.

The HGW algorithm is amenable for parallelism and coarse-grain pipelining; however, the large data buffers required to store *g*(*x*), *h*(*x*) and the pipelined computation of *r*(*x*) are identified as the most challenging aspects for a hardware implementation. In this sense, the solution proposed in [13] is not fully adequate for embedded scenarios as the memory resources are substantially high. In this paper, an FPGA-based memory resource efficient architecture that exploits parallelism and pipelining is presented. The goal is to achieve an optimized embedded implementation with a high throughput while reducing the dedicated on-chip Block Ram memory by an efficient utilization of the distributed memory resources available in current FPGA devices.

## 3. Proposed Architecture

The HGW algorithm in addition to modularity and regularity exhibits the following desirable properties for a hardware implementation [1]: (1) operations reducing to few comparator modules, (2) local and regular data and control flow requirements, and (3) inherent pipelining and multiprocessing features. The whole architecture is examined and its main components are described in detail in the following subsections.

### 3.1. Architecture Overview

**Figure 2:**
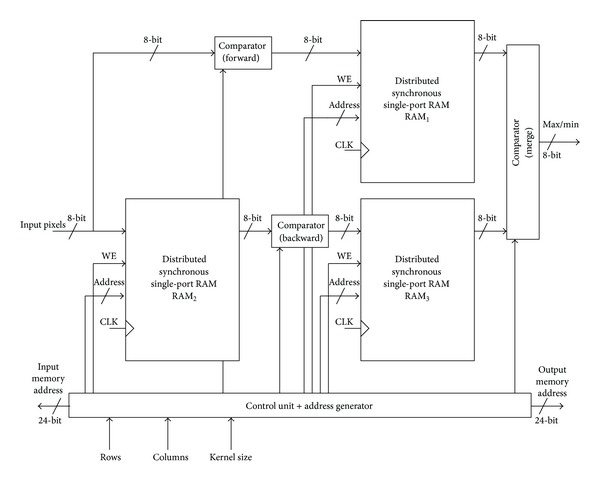
Block diagram of the proposed hardware architecture for running max/min filters based on the HWG algorithm and its main components.

 A block diagram of the hardware architecture for the HWG algorithm is shown in Figure 2. A set of three comparators are required for the internal computations, which are well mapped to the FPGA resources. The comparators are labeled as forward, backward, and merge to indicate to which processing step each comparator belongs to. The counter-based control unit synchronizes all data and control flow among modules in the architecture. Additionally, it generates the external memory addresses both to read data from the input memory and to store the processed data in the output memory according to image and kernel specifications. For the purpose of simplicity, just the main control signals are shown in Figure 2.

The major building blocks in the proposed architecture are memory units since the HGW algorithm is memory centric in the sense that more resources are required for internal storage than for computation. This is an example of the so-called systems on-chip that requires frequent sharing, communication, queuing, and synchronization among distributed functional units [15].

### 3.2. Memory Organization and Mapping

The architecture memory organization is based on the scheme used in [13], where three Block Ram modules were used. However, herein a different strategy is used to map logical memories to FPGA distributed memory such that function-level parallelism can be further exploited to improve scalability and performance. In [13], dual-port memories were used to ease the propagation and memory accesses. In addition, a block mirroring strategy was proposed to suppress the need for a complete backward scanning of the input stream. The mirroring scheme requires two Block Rams, RAM_2_ and RAM_3_, whose depth depends on the maximum supported kernel size. The third memory unit, RAM_1_, provides temporal storage for the computed values in the forward step and to synchronize the pipeline stages as will be explained in the following section.

In this paper, we use the memory resources distributed across the FPGA instead of the embedded Block Rams of unique size (i.e., 18 Kbit for Xilinx Spartan-6 devices). Some LUTs within each configurable logic block (CLB) optionally implement a 16 × 1-bit synchronous RAM which can be cascadable for deeper and/or wider memories [16]. Distributed RAM writes synchronously and reads asynchronously. This property is exploited in this work so as to avoid the use of dual-port memories. Furthermore, the address port either for single- or dual-port modes is asynchronous with an access time equivalent to a LUT logic delay.

### 3.3. Architecture Pipeline Scheme

**Figure 3:**
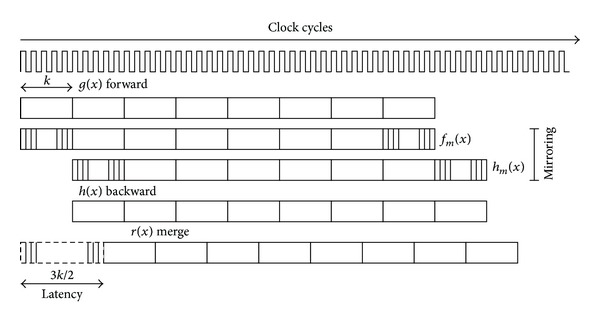
Pipeline scheme used in the HGW architecture. The computations and data flow are organized around a three-stage pipeline, forward, backward, and merge.

Figure 3 shows a high-level overview of the three-stage pipeline scheme used in the architecture in order to sustain a high output data rate. The internal memory requirements of the pipeline are provided by using low overhead on-chip memory, distributed synchronous single port rams, that alleviates the need of dual-port rams. The three memory units can be implemented very efficiently on FPGAs by taking advantage of concurrent synchronous writing and asynchronous reading since only streaming operations are required on windows of at most *k* elements.

The coarse-grain computational stages in the pipeline can be described as follows. In the first stage, two processing tasks are performed concurrently on the incoming data stream. The max value is propagated in a forward way and the stream values also undergo a reverse order arrangement in segments of size *k*. The second pipeline stage starts its operation after *k* clock cycles, and it performs the forward propagation of the previous mirrored segment and a backward mapping is also applied. The third stage starts the computations after the second stage completes the computation of *k*/2 output samples. Since the merge stage requires the data computed by the forward and backward stages becomes available, its operation must be delayed 3*k*/2 clock cycles after it can operate continuously on the *g*(*x*) and *h*(*x*) streams as shown in Figure 3. For synchronization purposes, the values of the forward stage must be delayed *k* clock cycles. This buffering is also implemented using a distributed synchronous single-port memory.

**Figure 4:**
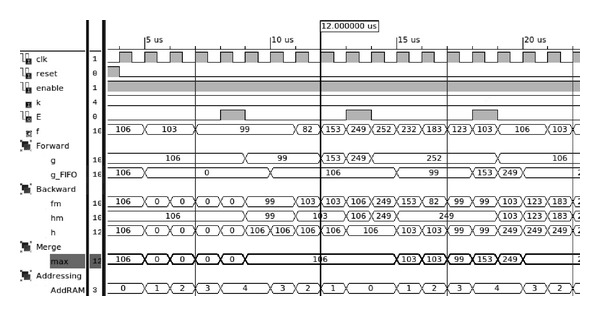
Timing diagram snapshot of the architecture functionality for running max filtering over the input pixel stream, *f*, using a kernel of size *k* = 5. The first output result is produced at 12 microseconds as indicated by the vertical line; then, results are produced on each clock cycle. The signal *AddRAM* shows the addresses generated through time used for accessing the synchronous single-port memories.

Figure 4 shows a time diagram of an 8-bit pixel stream *f* of an input image used to illustrate the operation of the architecture when a kernel of size *k* = 5 is used. A snapshot of the main signals *g* and *h* in the data flow and computation steps for the pipelined architecture are shown in the simulation assuming a clock frequency of 100 MHz. For simplicity, just two control signals *E* and *AddRAM* derived from the counter-based controller are shown. Note that *AddRAM* is generated by reverted address counters and used as addresses to write and read data in the distributed memory. Each stage is active for *k* consecutive clock cycles and the operation of adjacent stages are delayed for *k* clock cycles. Signal *E* indicates the time when a window of the input stream has been processed. As shown in Figure 4, each comparator, after being reset by *E*, is reused for another adjacent *k* window.

### 3.4. Parallelism Enhancement

**Figure 5:**
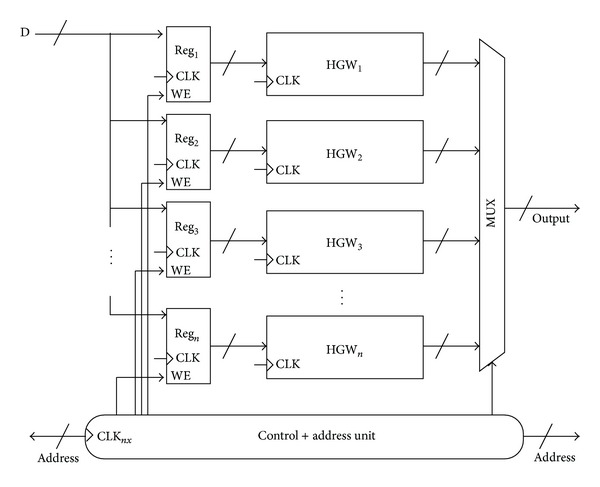
Organization of several HGW processing units to exploit function-level parallelism. The address generator unit works at a clock speed *n* times faster than the computational modules.

Because pipelining and parallelism are naturally supported by intrinsic resources of current FPGA devices, it is important to fully utilize these resources to improve performance. At a first level, the proposed architecture was divided into a set of simpler functional elements to carry out the internal computations in a pipelined fashion on the input stream. However, the performance for running max/min filters on two-dimensional signals using rectangular kernels can be improved if function-level parallelism is exploited. Thus, the HGW module can be replicated as much as possible and organized in a more parallel structure as shown in Figure 5, so as to process concurrently several input streams. In this sense, the number of HGW modules depends on the capacity of the target FPGA device and the actual memory organization that provides data. A set of registers is used to store data coming from the external memory. These registers provide parallel data access to the HGW modules. A multiplexer selects the results produced by the HGW modules and sends them to the output memory.

 To apply such parallel scheme for running max/min filters on images, it is assumed that the input image is scanned row by row starting from the upper-left corner sample. In addition, observing that memories can often operate much faster than the user's actual design, memory ports can be time multiplexed to increase the number of independent accesses. In such multipumping scheme [15], the memory system is clocked at a multiple of the main clock, providing the illusion of a multiple port memory. Multipumping brings an area reduction if the external memory speed is significantly higher than the required by the rest of the system. Since the number of required ports or the operating frequency is modest in the proposed design, the main benefit of multipumping is reducing the on-chip memory area at the expense of clock frequency.

## 4. Implementation and Experimental Results

In this section, experimental results of the FPGA implementation, the hardware resource utilization, and the performance evaluation of the proposed architecture are presented and discussed. 

### 4.1. FPGA Implementation

**Figure 6:**
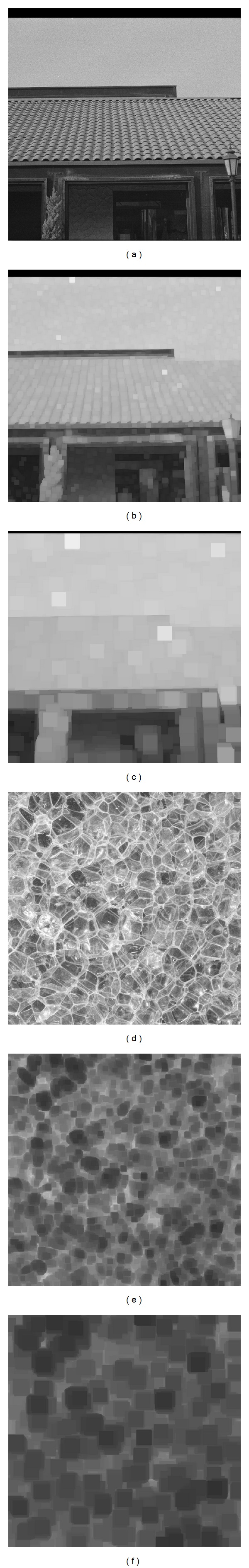
Examples of 1024 × 1024 test images used to validate the architecture functionality. ((a) and (d)) the input images and (b) max filter by 21 × 21, (c) max filter by 63 × 63, (e) min filter by 21 × 21, and (f) min filter by 63 × 63.

The Atlys FPGA board from Digilent Inc. has been used for prototyping and VHDL was used as the modeling language. Design parameters such as the kernel size, the image dimensions, and the number of parallel units are parameterizable, so they can be set to the appropriate values before synthesis for an optimized implementation. The architecture functionality has been validated on a set of test gray-level images from the Brodatz texture dataset.  Figure 6 shows two 1024 × 1024 test images used in the experiments and the results obtained produced by the architecture using rectangular kernels of different sizes, 21 and 63, for max and min filtering. Larger kernel sizes were also tested and validated but for space consideration are not presented here.

Table 1 shows the FPGA resource utilization and the maximum achievable frequency for three different instances of the architecture using 1, 2, and 4 HGW modules. The presented results are obtained from the reports generated by the Xilinx ISE 13.1 tool suite when the design is targeted to a Spartan-6 device. The entire HGW architecture pipeline fits easily into the device thanks to the use of distributed ram resources. Note that the three logic memory modules used in the design are mapped to LUTs in the FPGA device. Only 96 6-input LUTs are necessary to support any kernel size up to 255, that is, a 256 × 8 single-port distributed ram. The maximum clock frequency reported by the tool is 250 MHz for a single HGW module with less than one percent of usage of the target device. Thus, potentially a large number of HGW modules can be used without a considerable increase of resource utilization or speed degradation. The hardware resource utilization for a single HGW is similar to the proposed in [13] where 700 slices and 3 Block Rams of 18 Kbits were required. However, recall that authors used an FPGA technology relying on 4-input LUTs and in this work, the used target device natively supports 6-input LUTs; thus, a more compact implementation is expected. On the other hand, the use of distributed synchronous ram allows to replicate the HGW module so as to increase performance. A post-place-and-route simulation model was used to estimate the power consumption of the proposed architecture using the Xilinx XPower tool. The total power consumption of the 4-HGW design is 0.22 W, dynamic (0.18 W) plus quiescent (0.04 W) power.

### 4.2. Performance Evaluation

**Figure 7:**
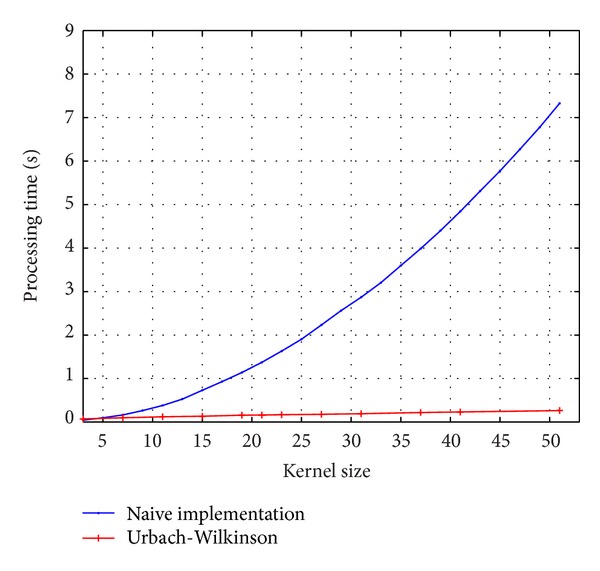
Processing time for the running max/min filters on an 2160 × 1440 input image with different kernel sizes for a straightforward implementation and the Urbach-Wilkinson algorithm.

In order to have a baseline for comparison, a straightforward implementation for min/max filtering was carried out in C programming language. Also, the Urbach-Wilkinson algorithm [17] is used for comparison purposes by using the source code provided by authors.  Figure 7 shows the computation times for these methods over 2160 × 1440 gray-scale images. The implementations were carried out on a MacBook Pro with an Intel Core i7 2.66 GHz processor and 4 GB main memory in ANSI C without multithreading and compiled using gcc with O3 optimization flag set. The computation time for the straightforward implementation grows prohibitively large, not being suitable for real-time performance.

**Figure 8:**
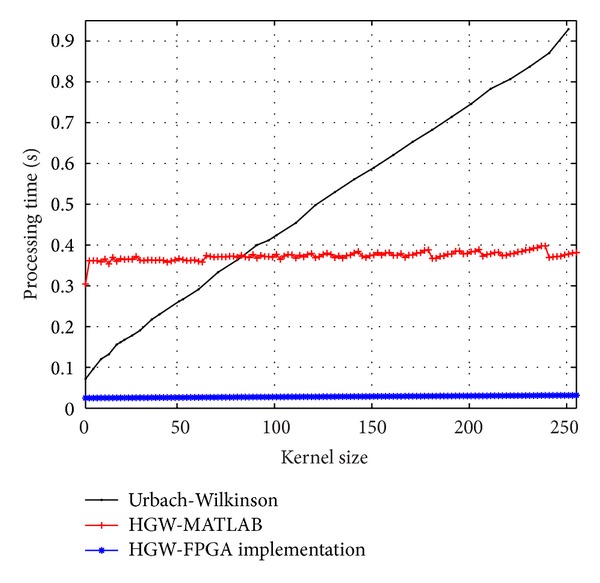
Processing time for the running max/min filters on an 2160 × 1440 input image with different kernel sizes using a single HGW module in the proposed architecture clocked at 250 MHz. The processing time required for the Urbach-Wilkinson algorithm and the MATLAB implementation of the HGW algorithm is also shown for comparison.

Figure 8 shows the near constant processing time, around 25 milliseconds, required for the architecture to filter a 2160 × 1440 input image for different kernel sizes. Recall that the architecture must operate twice on the input image since it uses kernel decomposition. A single HGW module clocked at 250 MHz processes row by row and then column by column. The architecture takes 3*k*/2 clock cycles to produce the first result.

The processing time required for the MATLAB erosion/dilation optimized implementation is used for comparison purposes. Though it is not a parallel implementation, it forms a useful comparison baseline for this work as it uses the HGW algorithm and implements kernel decomposition. Figure 8 shows that the proposed architecture is faster than the MATLAB optimized implementation, above 10x, with a deterministic response, and also outperforms the Urbach-Wilkinson implementation. The very low resource utilization makes the architecture suitable for embedded applications in low-cost FPGA devices with similar performances as efficient implementations in graphical processing units (GPUs) but with much lower power consumption.

**Figure 9:**
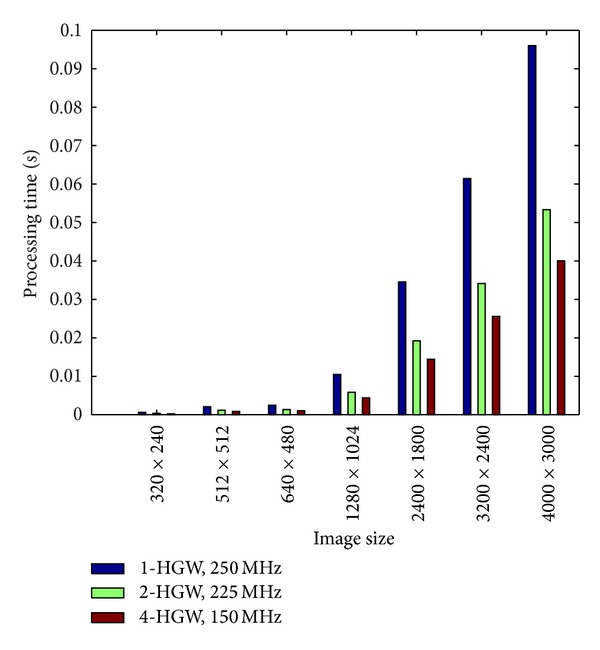
Processing time for different image sizes using three different degrees of parallelism.

 The architecture is able to process images of different sizes and it can be easily extended to improve its performance by the replication of the HGW module.  Figure 9 shows the processing times required for max/min filtering for different image sizes and for three degrees of parallelism using 1, 2, and 4 HGW modules clocked at 250 MHz, 225 MHz, and 150 MHz, respectively. Note that when 4 HGW instances are used, it is still possible to achieve more than standard real-time performance, 30 frames per second, even for high-resolution images. 

### 4.3. Application on Local Adaptive Binarization

 According to the results, the proposed architecture is suitable to be applied in embedded applications thanks to its real-time performance, low resource utilization, and low power consumption. In this section, a further application of the architecture for image binarization is presented. Image binarization converts gray-level or color images into binary ones in order to distinguish objects from background by finding and applying an appropriate threshold for image pixels. In document image analysis, the main goal is to extract printed characters through optical character recognition (OCR) to analyze relevant textual information from document images from sources such as books, magazines, forms, or newspapers [18]. Local thresholding methods find a threshold for each image pixel based on local characteristics and statistics of pixels within a neighborhood centered around a given pixel [19, 20]. Motivated by the advantages of local adaptive thresholding and by the challenge to handle efficiently the computational cost and the memory bandwidth, proposed architecture has been applied to accelerate computations for the Bernsen algorithm.

In the Bernsen algorithm [21], for each pixel of the original image with gray level *I*(*x*, *y*)∈[0,255], the local threshold, *T*(*x*, *y*), is set at the midrange value, which is the mean of the minimum and maximum gray level values in a given neighborhood:

(5) $$\begin{array}{c} T\left(x,y\right)=\frac{1}{2}\left[I_{\max}\left(x,y\right)+I_{\min}\left(x,y\right)\right]. \end{array}$$

If the contrast *c*(*x*, *y*) = *I*_max⁡_(*x*, *y*) + *I*_min⁡_(*x*, *y*) in the neighborhood is below a given threshold *k*, then it is assumed that the neighborhood consists only of one class, foreground or background, depending on the value of the threshold. Each pixel(*x*, *y*) is classified as an object pixel (indicated by value 1) or a background pixel (indicated by value 0) according to the following equation:

(6) $$\begin{array}{c} b\left(x,y\right)=\{\begin{array}{cc} 1, & \text{if}\;I\left(x,y\right)<T\left(x,y\right),c\left(x,y\right)>k,\\ 0, & \text{otherwise}. \end{array} \end{array}$$

**Figure 10:**
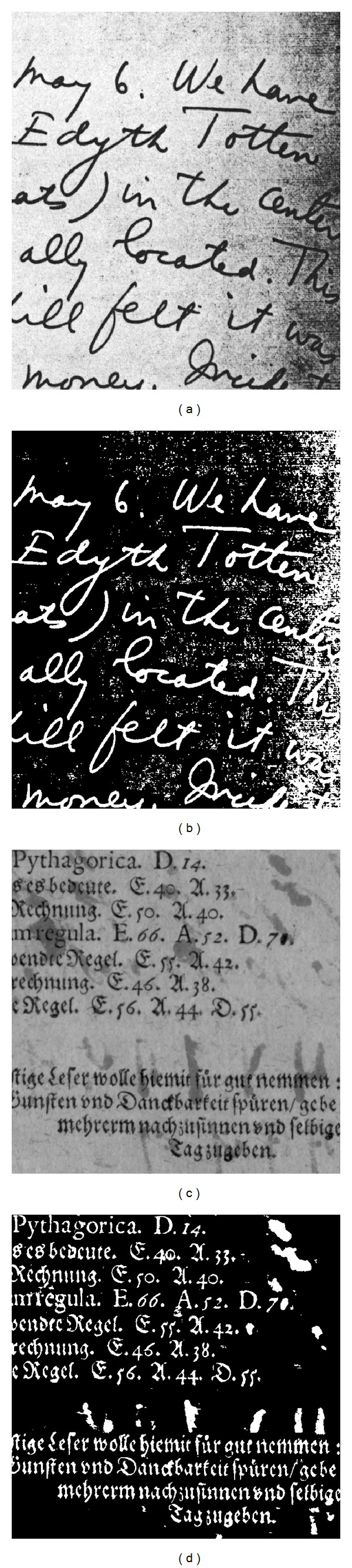
Test images used to show the applicability of the architecture for local adaptive thresholding. ((a) and (c)) The input images; ((b) and (d)) thresholded images by using the Bernsen algorithm with a 31 × 31 window and *k* = 60.

The major part of the computations involved in this method is the calculation of local maximum and local minimum. Thus, the Bernsen algorithm fits well the proposed architecture to speed up computations by using two HGW modules working in parallel.  Figure 10 shows two input images and the corresponding binarized ones using a window of 31 × 31 and *k* = 60. The processing time to binarize a 1024 × 1024 image with this window size is 8.4 milliseconds, 120 frames per second, at a maximum clock frequency of 250 MHz, when the architecture is targeted to a Spartan-6 device. This yields a throughput over 14 Gpixels/second enough for real-time image processing. Recall, however, that real time is a context relative measure.

## 5. Conclusions

An efficient implementation of a fast algorithm for arbitrary length max/min filters has been presented. The proposed architecture is very regular and scalable with a good resource-performance tradeoff suitable to be embedded in low-cost FPGA devices. The proposed design takes advantage of distributed memory resources available in current programmable devices without introducing a high-performance penalty. However, for very large kernel sizes, the area for distributed memory increases rapidly and the operating frequency might drop significantly. This motivates the use of the specialized Block Rams as a more efficient solution. The results show that the proposed implementation could achieve the same throughput with less amount of memory resources compared to the previously reported solution. The architecture, when targeted to a Spartan-6 device, can compute a max/min running filter over a 1024 × 1024 image with a kernel size up to 255 in 8.4 milliseconds at a maximum clock frequency of 250 MHz. This performance is sufficient for real-time full-HD video processing. The progress of high resolution image applications on embedded systems requires reviewing existing solutions under this context and proposing hardware accelerators to potentially provide practical, compact, and low power solutions. For future work, it is planned to analyze in detail the power consumption of the proposed implementation, extend further the applicability of the proposed hardware to local adaptive thresholding, and to implement specialized operators in gray-level image morphology.

## Figures and Tables

**Table 1:** Summary of the hardware resource utilization for the proposed architecture targeted to a Xilinx Spartan-6 LX45 device for different number of instances of the HGW module.

Resource utilization (total available)	1-HGW	2-HGW	4-HGW
Slice registers (54576)	75	150	216
Slice LUTs (27288)	258	566	910
LUTs used as logic (27288)	159	368	518
LUTs as memory (6408)	96	192	384
RAMB16BWERs (116)	0	0	0
RAMB8BWERs (232)	0	0	0
Maximum frequency	250 MHz	232 MHz	215 MHz
